# Reddit financial image post sentiment dataset

**DOI:** 10.1016/j.dib.2022.108759

**Published:** 2022-11-17

**Authors:** Alexander Fottner, Yarema Okhrin, Jonathan Pfahler, Julian Wustl

**Affiliations:** a Department of Statistics, Faculty of Business and Economics, University of Augsburg, Universitaetsstr. 16, 86159 Augsburg, Germany; b Department of Computer Science and Mathematics, Munich University of Applied Sciences, Lothstr. 34, 80335, München, Germany

**Keywords:** Sentiment analysis, Image sentiment, Meme stocks, Social media

## Abstract

The dataset presented in this paper consists of sentiment information extracted from image and text data of financial subreddit posts. Members of these subreddits post about their trading behavior, express their opinions, and discuss capital market trends. Their posts contain sentiment information on financial topics as well as signaling information on trading decisions. Frequently, members post screenshots of their portfolios from their mobile broker apps. We collected the posts, processed them to extract sentiment scores using various methods, and anonymized them. The dataset consists therefore not of any content from the posts or information about the author, but the processed sentiment information within the post. Further financial tickers mentioned in the posts are tracked, such that the effect of sentiment in the posts can be attributed to financial products and used in the context of financial forecasting.

The posts were collected using the Reddit [2] and Pushshift APIs [3] and processed using an Amazon Web Services architecture. A fine-tuned MobileNets artificial neural network [4] was used to classify images into four distinct categories, which had been determined in a preliminary analysis. The categories included *classical memes, number posts* (e.g. screenshots of mobile broker portfolios), *text posts* (e.g. screenshots from twitter) and *chart posts* (e.g. other financial screenshots, such as charts). The reason for the classification of images into the four categories is that the images are so inherently different, that different extraction methods had to be applied for each category. OCR – methods [5] were used to extract text from images. Custom methods were applied to extract sentiment and other information from the resulting text. The data [1] is available on a 20-minute basis and can be used in many areas, such as financial forecasting and analyzing sentiment dynamics in social media posts.

**Specifications Table**

**Table:** 

Subject	Financial Markets and Institutions
Specific subject area	Financial sentiment from social media posts, especially, the area of sentiment extraction from image data.
Type of data	Three CSV files: - meta_time_series.csv covers the time series of meta information of posts. - features.csv covers the (sentiment) information extracted from each post. - comments.csv covers the (sentiment) information extracted from the comments associated with each post.
How the data were acquired	Extraction via API calls from financial subreddits and subsequent feature extraction using a custom-built feature engineering pipeline on an architecture using a cloud computing service provider.
Data format	Mixed (raw and preprocessed)
Description of data collection	Raw social media posts were collected via API calls from reddit.com. The data was processed using a custom-built feature engineering pipeline on an architecture using a cloud computing service provider. We anonymized the data by hashing the user id for each post.
Data source location	The data was collected from the following publically available subreddits of reddit.com: *r/finance, r/financialindependence, r/forex,r/gme, r/investing, r/options, r/pennystocks, r/personalfinance,r/robinhoodpennystocks, r/securityanalysis, r/stockmarket, r/stocks,r/wallstreetbets.*
Data accessibility	Repository name: Mendeley Data, Data identification number: 10.17632/b6ns6d8xv3.3 Direct URL to data: https://data.mendeley.com/datasets/b6ns6d8xv3/3

## Value of the Data

• The data provides quantitative sentiment extracted from text and images on finance related social media posts on reddit. The data can be aggregated on a 20-minute, hourly or daily basis and be used in time series analyses.
• Further, the dynamics and changes in sentiment can be analyzed over time and across posts, which is relevant for the field of sentiment analysis.
• The features can be further used in the context of Machine Learning prediction. Since financial tickers are often included for posts, one can analyze the influence of changes in sentiment on stocks.
• Generally, the data can be used as additional data to conventional datasets in the context of stock price prediction.
• Further, the extensive time series information of posts allows the research of dynamics, that drive the popularity of memes and other social media posts and determine the factors that makes posts go viral.
• As the data set consists of sentiment extracted from financial subreddit posts, it allows for analyses in the context of behavioral finance in respect to the members of such forums. Further, educators can use the variety of features to demonstrate all kinds of models and methods in the fields of Machine Learning and Data Mining.

## 1 Objective

The data was generated to allow the investigation of the relationship between sentiment contained in social media posts on Reddit and movements on the financial markets. The datasets convers not only sentiment that is extracted from textual data, but images as well, which has not been done so far in this context.

## 2 Data Description

The data [1] consists of sentiment information extracted from social media posts of financial subreddits. We applied custom-written methods to images and text to extract sentiment values and create the features in the provided data set files. We collected the data to extend the research capacities in the field of sentiment analysis in financial forecasting. In particular, we aim to facilitate the area of sentiment extraction from images. With this paper we provide the three csv files *features.csv, comments.csv* and *meta_time_series.csv*, containing different parts of the data. The datasets start on different dates, but end on the same date and the shared range covers more than 3 months. The *features.csv* ranges from October 1^st^ 2021 to February 25^th^ 2022, *the comments.csv* ranges from November 12^th^ 2021 to February 25^th^ 2022 and the *meta_time_series.csv* ranges from November 14^th^ to February 25^th^ 2022. The reason each data set has different start dates is because each data set was created by a different feature of the pipeline and they were ready to launch at different times. We chose to begin implementing each feature as early as possible to collect the largest amount of data for each dataset.

The file *features.csv* contains the static sentiment features that were extracted from the content of the posts. Although the observations in this file have time stamps, the file does not contain time series information itself, since the content of a post usually does not change over time.

This is different for *meta_time_series.csv*, which stores meta-information of a post, since variables such as the number of comments changes drastically over time. Therefore, *meta_time_series.csv* contains the time series for the meta-information of each post over the lifetime of a post. To map the static information from the *features.csv* on the data in meta_time_series.csv, the variable *submission_id* can be used. Due to privacy reasons, the submission ids were hashed. This way, they can still be used for the unification of the data in the three files, but not be used for identification of the post's author. Lastly, *comments.csv* contains features from sentiment extraction methods applied to the comments for each post. The data in *comments.csv* also contains time series information since the number of comments below a post might change over time. The methods were applied on all comments below a post and produce an aggregated value. The resulting value can change over time when new comments are posted for a post.

## 3 Experimental Design, Materials and Methods

The approach we followed to create the dataset consists of several steps. First, the raw data was collected from Reddit using API calls on a 20-minute basis. We tracked static variables, such as the ones derived from the content within the post, as well as non-static variables, such as the ones derived from comments, which constantly change. The posts were retrieved using the Reddit API [2] as well as the Pushshift API [3]. Subsequently, they were processed using a custom feature extraction pipeline running on Amazon Web Services servers. A Mobile Nets artificial neural network [4] was trained to classify the images contained in posts into four categories, since the images were so inherently different in the structure of their contents that different methods for sentiment extraction needed to be applied. We use several custom functions to create sentiment variables from the image and textual information in the posts as well as the title and meta-information, according to the descriptions in Table 1-3 above. The output of this pipeline are the final features containing different forms of sentiment. The time series sequences contained in the data are rather short since posts are tracked for as long as they are relevant and never longer than for 24 hours. Some of the variables include outliers. The reason for this is, for example, that the author of a post might exaggerate or even just posts an unrealistically high number (e.g. of realized percentage gains) as a joke. Our methods can not filter for such scenarios. We chose not to exclude outliers from the dataset and provide the raw data and leave it to the individual researcher using this dataset, to decide on how to deal with this issue.

**Table 1:** Features.

Variable	Type	Description	Source/Engineering
bear_score	float	Degree of bearish sentiment in a post.	Bearish positions are identified using a custom list for keywords of bearish sentiment^1^. The number of bearish words in a post is divided by the sum of the numbers of all bearish and bullish keywords within the post.
num_bearish	integer	Number of bearish mentions in a post.	As for the bear_score, bearish positions are identified using a custom list for keywords of bearish sentiment^2^. The value is the number of bearish positions within the post.
bull_score	float	Degree of bullish sentiment in a post.	Bullish positions are identified using a custom list for keywords of bullish sentiment. The number of bullish words in a post is divided by the sum of the numbers of all bearish and bullish keywords within the post.
num_bullish	integer	Number of bullish mentions in a post.	As for the bull_score, bullish positions are identified using a custom list for keywords of bearish sentiment. The value is the number of bearish positions within the post.
is_long	binary	Indicates, whether a post contains long positions.	Equals 1, if the post contains *long* positions, else 0. If there are any long positions mentioned in the post, the value is 1, else 0. A long position is identified only on images of portfolio position or a market order.
colour_scheme	categorical	Describes whether the color scheme in the image suggests predominantly positive or negative sentiment.	If there are more green than red pixels, the entry is "positive", if there are more red than green pixels it is "negative", else it is "nan". In images of portfolio positions green pixels suggest gains, red pixels suggest losses.
flair	categorical	Flair attached to the post.	Meta - information extracted directly from each post.
gain_score_per	float	*Gain* sentiment from positive percentage values in a post.	Aggregate of all positive percentage values in a post. A lot of positive percentage values indicate a higher gain sentiment.
gain_score_real	integer	*Gain* sentiment from positive absolute values in a post.	Aggregate of all positive absolute values in a post. A lot of positive absolute values indicate a higher gain sentiment.
type_post_content	categorical	Type of content in a post.	A pre-trained MobileNets image classification model is fine-tuned to classify each image in a post into one of four categories. The categories are "number", "chart", "text", "meme" images. Further, if the post contains a gif, text or link instead, "gif", "text", "link" is given as value. Else "unknown" is given.
loss_score_per	float	Aggregate of all negative percentage values in a post. A lot of negative percentage values indicate a higher *loss* sentiment.	Negative percentage values are aggregated.
loss_score_real	integer	Aggregate of all negative absolute values in a post. A lot of negative absolute values indicate a higher *loss* sentiment.	Negative absolute values are aggregated.
normal_sentiment_ weighted	float	Value for overall positive or negative sentiment in the text of a post, ranging between -1 (most negative) and 1 (most positive).	Text in images and titles is evaluated using the sentiment classification model VADER [6]. Additional custom weights are introduced to weigh sentiments based on group-specific keywords^3^ which are used by the communities in the considered subreddits.
normal sentiment_ score_positive	float	Value for the degree of positive sentiment, ranging between 0 (no positive) and 1 (most positive).	Text in images and titles is evaluated using the sentiment classification model VADER for positive sentiment only. Additional custom weights are introduced to weigh sentiments based on group-specific keywords which are used by the communities in the considered subreddits.
normal_sentiment_ score_negative	float	Value for the degree of negative sentiment, ranging between 0 (no negative) and 1 (most negative).	Text in images and titles is evaluated using the sentiment classification model VADER for negative sentiment only. Additional custom weights are introduced to weigh sentiments based on group-specific keywords which are used by the communities in the considered subreddits.
posted_at	string	Timestamp when post was posted.	Meta - information derived directly from each post.
is_short	float	Equals 1, if the post contains *short* sentiment, else 0.	If there are any short positions mentioned in the post, the value is 1, else 0. A long position is identified only on images of portfolio position or a market order.
num_shares_mentions	integer	Number of times the word *shares* is mentioned in a post.	Number of times the word *shares* is mentioned in a post.
social_media_type	categorical	Kind of post - either "twitter", "reddit" or "unknown".	If the post is a repost from twitter, it will be classified as "twitter", if it is originally from reddit it is classified as "reddit", else "unknown".
submission_id	string	Unique identifier for each post (anonymized)	Meta - information derived directly from each post via API call.
ticker	list	List of mentioned tickers.	Tickers in post were identified using a keyword list and regular expressions searching for a $ sign in front of strings.
timestamp	string	Time when the post was pulled via API call.	Meta - information derived directly from each post via API call.

1 keywords of bearish sentiment: [‘short’, ‘sell’, ’lost’, ‘loss’, ‘down’, ‘put’, ‘drop’ ,’bear’, ‘red’, ‘leave’]

2 keywords of bullish sentiment: = [‘call’, ‘buy’, ‘bull’, ‘moon’, ‘long’, ‘win’, ‘gain’, ‘going up’, ‘rocket’, ‘long term’, ‘green’, ’yolo’]

3 Additional custom weights: {'citron': -4.0, 'hidenburg': -4.0,'moon': 4.0, 'highs': 2.0,'mooning': 4.0, 'long': 2.0, 'short': -2.0, 'call': 4.0, 'calls': 4.0, 'put': -4.0, 'puts': -4.0, 'break': 2.0, 'tendie': 2.0, 'tendies': 2.0, 'town': 2.0, 'overvalued': -3.0, 'undervalued': 3.0, 'buy': 4.0, 'sell': -4.0, 'gone': -1.0, 'gtfo': -1.7, 'paper': -1.7, 'bullish': 3.7, 'bearish': -3.7, 'bagholder': -1.7, 'stonk': 1.9, 'green': 1.9, 'money': 1.2, 'print': 2.2, 'rocket': 2.2, 'bull': 2.9, 'bear': -2.9, 'pumping': -1.0, 'sus': -3.0, 'offering': -2.3, 'rip': -4.0, 'downgrade': -3.0, 'upgrade': 3.0, 'maintain': 1.0, 'pump': 1.9, 'hot': 1.5, 'drop': -2.5, 'rebound': 1.5, 'crack': 2.5}

**Table 2:** Comments.

Variable	Type	Description	Source/Engineering
comment_long	float	Average number of *long* sentiment in all comments of a post.	See is_long in Table 1.
comment_gain	float	Number of comments of a post with positive values posted divided by the total number of comments of the post.	See gain_score_real in Table 1.
comment_loss	float	Number of comments of a post with negative values posted divided by the total number of comments of the post.	See loss_score_real in Table 1.
comment_short	float	Average number of *short* sentiment in all comments of a post.	See is_short in in Table 1.
comment_shares_reference	integer	Number of *shares* mentioned in all comments of a post.	Number of times the word *shares* is mentioned in all comments under a post.
comment_total_score	float	Sum of scores of all comments of a post.	Meta - information derived directly from each post via API call.
comment_normal_ sentiment_negative_ weighted	float	Average value of VADER negative sentiment scores multiplied with the comments score of all comments of a post.	Text in comments is evaluated using the sentiment classification model VADER for negative sentiment only. Additional custom weights are introduced to weigh sentiments based on group-specific keywords which are used by the communities in the considered subreddits. The resulting score is weighted with the number of comments of the associated post.
comment_normal_ sentiment_positive_ weighted	float	Average value of VADER positive sentiment scores multiplied with the comments score of all comments of a post.	Text in comments is evaluated using the sentiment classification model VADER for positive sentiment only. Additional custom weights are introduced to weigh sentiments based on group-specific keywords which are used by the communities in the considered subreddits. The resulting score is weighted with the number of comments of the associated post.
comment_total_sentiment	float	Average value of VADER absolute sentiment scores multiplied with the comments score of all comments of a post.	Text in comments is evaluated using the sentiment classification model VADER for both positive and negative sentiment. Additional custom weights are introduced to weigh sentiments based on group-specific keywords which are used by the communities in the considered subreddits. The resulting score is weighted with the number of comments of the associated post.
timestamp	string	Time when the post was pulled via API call.	Meta - information derived directly from each post via API call.
posted_at	string	Time, when the comment is posted.	Meta - information derived directly from each post via API call.
submission_id	string	Unique identifier for the corresponding post (anonymized).	Meta - information derived directly from each post via API call.

**Table 3:** Meta data.

Variable	Type	Description	Source/Engineering
crossposts	integer	Number of reposts of the post by other Reddit users.	Meta - information derived directly from each post via API call.
num_comments	integer	Number of comments associated with the post.	Meta - information derived directly from each post via API call.
score	integer	The number of upvotes for the submission.	Meta - information derived directly from each post via API call.
upvote_ratio	float	The percentage of upvotes from all votes on the submission	Meta - information derived directly from each post via API call.
timestamp	datetime object	Time when post was pulled via API call.	Meta - information derived directly from each post via API call.
submission_id	string	Unique identifier for each post (anonymized).	Meta - information derived directly from each post via API call.

**Table 4:** Summary statistics - features.

	mean	std	min	25%	50%	75%	max
bear_score	1.12E-01	2.96E-01	0	0	0	0	1.00E+00
num_bearish	2.07E-01	1.15E+00	0	0	0	0	7.70E+01
bull_score	8.93E-02	2.62E-01	0	0	0	0	1.00E+00
num_bullish	0.603	2.59E+00	0	0	0	0	1.95E+02
gain_score_per	1.29E+07	4.22E+09	0	0	0	0	1.99E+12
gain_score_real	4.64E+21	2.26E+24	0	0	0	0	1.10E+27
loss_score_per	1.53E+05	5.72E+07	0	0	0	0	2.76E+10
loss_score_real	4.18E+13	2.04E+16	0	0	0	0	9.92E+18
normal_sentiment_weighted	0.247	5.32E-01	-1	0	0.2732	0.7184	1
normal_sentiment_score_negative	0.119	1.61E-01	0	0	0.06	0.175	1
normal_sentiment_score_positive	0.0952	0.124	0	0	0.061	0.15	1
num_shares_mentions	0.0963	0.898	0	0	0	0	105

**Table 5:** Summary statistics - Comments.

	mean	std	min	25%	50%	75%	max
comment_long	2.0761	17.5819	0	0	0	1	5248
comment_gain	0.0173	0.1173	0	0	0	0	4.3333
comment_loss	0.0029	0.0372	0	0	0	0	2
comment_short	0.9406	9.4606	0	0	0	0	1766
comment_shares_reference	0.7377	11.8022	0	0	0	0	1693
comment_total_score	149.4535	769.0452	0	9	25	81	40157
comment_normal_sentiment_negative_weighted	0.1050	0.4003	0	0.0427	0.077	0.1093	166.9245
comment_normal_sentiment_positive_weighted	0.2054	0.7114	0	0.1171	0.151	0.1943	224.8655
comment_total_sentiment	0.2520	1.4103	0	0.0537	0.1527	0.2818	1079.3921

**Table 6:** Summary statistics - meta data.

	mean	std	min	25%	50%	75%	max
crossposts	0.05	0.3532	0	0	0	0	37
num_comments	34.5141	344.2240	0	4	9	20	33762
score	196.04	1024.7801	0	3	14	63	83576
upvote_ratio	0.7783	0.1969	0	0.67	0.83	0.94	1

The categorical variable *flair* was excluded from the summary statistics of Table 7, as there are 254 unique values and a breakdown of the distribution of observations over these categories is not reasonable.

**Table 7:** Summary statistics - categorical variables.

Categories of social_media_type	Number of observations
unknown	229201
Reddit	1621
Twitter	6411

Categories of colour_scheme	Number of observations
unknown	158742
negative	64113
positive	14378

Categories of is_short	Number of observations
0	229435
1	7798

Categories of is_long	Number of observations
0	219721
1	17512

## Ethics Statements

The data is based on publicly available social media posts, which have been processed in such a way, such that they do not contain any personal data or copyrighted material. Further the data is fully anonymized. Reddit's data redistribution policies were complied with.

## CRediT Author Statement

**Pfahler, Jonathan:** Conceptualization, Methodology, Writing- Original draft preparation, Writing- Reviewing and Editing. **Wustl, Julian:** Software, Programming. **Fottner, Alexander:** Data Preprocessing, Writing- Original draft preparation, Writing- Reviewing and Editing. **Okhrin, Yarema:** Supervision.

## Declaration of Competing Interest

The authors declare that they have no known competing financial interests or personal relationships that could have appeared to influence the work reported in this paper.

## Data Availability

Reddit financial image post sentiment dataset (Original data) (Mendeley Data)
